# Sex-Biased miRNAs in Gonad and Their Potential Roles for Testis Development in Yellow Catfish

**DOI:** 10.1371/journal.pone.0107946

**Published:** 2014-09-17

**Authors:** Jing Jing, Junjie Wu, Wei Liu, Shuting Xiong, Wenge Ma, Jin Zhang, Weimin Wang, Jian-Fang Gui, Jie Mei

**Affiliations:** 1 College of Fisheries, Key Laboratory of Freshwater Animal Breeding, Ministry of Agriculture, Freshwater Aquaculture Collaborative Innovation Center of Hubei Province, Huazhong Agricultural University, Wuhan, China; 2 State Key Laboratory of Freshwater Ecology and Biotechnology, Institute of Hydrobiology, Chinese Academy of Sciences, University of the Chinese Academy of Sciences, Wuhan, China; Sun Yat-sen University, China

## Abstract

Recently, YY super-male yellow catfish had been created by hormonal-induced sex reversal and sex-linked markers, which provides a promising research model for fish sex differentiation and gonad development, especially for testis development. MicroRNAs (miRNAs) have been revealed to play crucial roles in the gene regulation and gonad development in vertebrates. In this study, three small RNA libraries constructed from gonad tissues of XX female, XY male and YY super-male yellow catfish were sequenced. The sequencing data generated a total of 384 conserved miRNAs and 113 potential novel miRNAs, among which 23, 30 and 14 miRNAs were specifically detected in XX ovary, XY testis, and YY testis, respectively. We observed relative lower expression of several miR-200 family members, including miR-141 and miR-429 in YY testis compared with XY testis. Histological analysis indicated a higher degree of testis maturity in YY super-males compared with XY males, as shown by larger spermatogenic cyst, more spermatids and fewer spermatocytes in the spermatogenic cyst. Moreover, five miR-200 family members were significantly up-regulated in testis when treated by 17α-ethinylestradiol (EE2), high dose of which will impair testis development and cell proliferation. The down-regulation of miR-141 and 429 coincides with the progression of testis development in both yellow catfish and human. At last, the expression pattern of nine arbitrarily selected miRNAs detected by quantitative RT-PCR was consistent with the Solexa sequencing results. Our study provides a comprehensive miRNA transcriptome analysis for gonad of yellow catfish with different sex genotypes, and identifies a number of sex-biased miRNAs, some of that are potentially involved in testis development and spermatogenesis.

## Introduction

microRNAs (miRNAs), a class of small non-coding RNAs (∼18–26 nt), have been known to be involved in mRNA degradation and post-transcriptional repression [1]. Most mature miRNA sequences are conserved among fish, amphibians, birds and mammals[2]. miRNAs have been revealed to play important roles in many biological processes, such as tissue development, cell proliferation and differentiation [3]. In vertebrates, a subset of miRNAs, such as miR-430 and miR-196 are specifically expressed and functioning during early embryonic development [4], [5]. Fish miR-430 regulates early primordial germ cell development by regulating *sdf1a*, *cxcr7*, *TDRD7*, *nanos1* and *c1q-like* expression [6]–[9]. In adults of chicken and cattle, some miRNAs have been identified abundantly expressed in gonadal tissues [10], [11]. Let-7 regulates ageing of the Drosophila testis stem-cell niche by targeting IGF-II messenger RNA binding protein [12]. However, the regulatory and functional roles of miRNAs in gonad development have not been clear in teleosts yet.

In aquaculture, many fish species display significantly different growth rate between male and female. For example, in yellow catfish (*Pelteobagrus fulvidraco*), Nile tilapia (*Oreochromis niloticus*), African catfish (*Clarias gariepinus*), and channel catfish (*Ictalurus punctatus*), male exhibits much faster growth rate than female sibling [13]–[16]. While female growth much faster than male in some other aquaculture fishes, such as gibel carp (*Carassius auratus gibelio* Bloch), rainbow trout (*Oncorhynchus mykiss*) and half-smooth tongue sole (*Cynoglossus semilaevis*) [17]–[19]. The productions of mono-sex groups of fish can be accomplished by sex-reversal technology and subsequently improve fish production. Recently, YY super-male yellow catfish was successfully created by crossing XY male with hormonal-induced XY female, and then identified by sex-linked SCAR markers [20]–[22]. However, the molecular mechanism of gonad development is unknown in yellow catfish.

The miRNA expression profile in male and female gonad of yellow catfish has not been explored. Here, we performed deep sequencing using solexa technology on 3 types of gonad (XX females, XY males and YY males). Also, we investigated miRNA expression using quantitative real-time PCR (RT-qPCR). We found sexually dimorphic expression of many miRNAs, some of which have also been observed to have a sex-dependent expression pattern in other vertebrates. Our results indicate a wide conservation of miRNAs in teleost and suggest their possible roles in vertebrate testis development.

## Materials and Methods

### Fish samples

Experiments were performed on one-year-old yellow catfish individuals (four females, four males and three YY super-males) with the same age and under the same culturing conditions. Their sex was confirmed by histological analysis and PCR with sex-linked primers as described previously [23]. The males and super-males are of the same size and weight. Experimental protocols used here were approved by the institution animal care and use committee of Huazhong Agricultural University. Hematoxylin and eosin (HE) staining was performed according to previously described [24].

### RNA isolation, small RNA library preparation and sequencing

Gonad tissues were taken from each individual of 4 XX females, 4 XY males, 3 YY super-males. To prevent potential cell contamination, the gill of healthy yellow catfish was cut to remove most of the blood, and the gonad tissues were washed with fresh PBS for three times. Total RNA was isolated from each sample by a Qiagen miRNeasy Mini Kit (Qiagen, USA) and treated by RNase-Free DNase to eliminate potential DNA contamination, and then standardized to a concentration of 1 µg/µL. RNA from the same type tissues was combined into one RNA pool, whose quality and quantity was measured with NanoDrop 2000 and Agilent 2100 Bioanalyzer. Three small RNA libraries were constructed with these three RNA pools using the TruSeq Small RNA Sample Preparation Kits. Briefly, total RNA was ligated with a 3′ RNA adaptor and then with a 5′ RNA adaptor. PCR amplification after reverse transcription was performed to enrich the fragments that had adaptors on both ends. Subsequently, the cDNA constructs were purified and enriched with 6% denaturing polyacrylamide gel electrophoresis to isolate the expected size fractions (∼140–155 bp) and eliminate dimerized adaptors, unincorporated primers and primer dimer products. The quality of three libraries was assessed on a Bio-analyzer and confirmed by a narrow normal distribution centered around the expected size, indicating that there is no contamination of other length of sequence. Finally, these three RNA libraries were sequenced using an Illumina/Solexa Genome Analyzer at Shanghai OE Biotech Company.

### Sequencing data analysis

Raw reads obtained from Solexa sequencing were processed obtain clean reads by summarizing data production, evaluating sequencing quality. After removing adapter sequences and low-quality reads, high-quality reads between 16 and 30 nt in length were processed for bioinformatics analysis with a proprietary software package: ACGT101-miR v4.2 (LC Sciences, Houston, USA). The sequencing sequences (sequ-seqs) were searched against pre-miRNA (pre-miR) and mature miRNA (miR) sequences from selected species (Deuterostoma) listed in the miRBase v20.0 (http://www.mirbase.org/). Reads mapped to mRNA in NCBI Genebank, non-coding RNAs (rRNA, tRNA, snoRNA, snRNA and others) in Rfam (http://rfam.janelia.org) and repetitive sequence elements in RepBase (www.girinst.org/repbase) were removed before further analysis. The retain reads were aligned to *Danio rerio* genome and EST sequences as primary source of reference since the *P. fulvidraco* genome was not available. Flanking sequences from each mapping locus were subjected to secondary structure analysis using RNAfold (http://rna.tbi.univie.ac.at/cgi-bin/RNAfold.cgi) with the default folding criteria. The detailed mapping process was performed as previously described [25]. Then, the nohit reads were blasted against the piRNA database download from piRNA cluster-database (http://www.uni-mainz.de/FB/Biologie/Anthropologie/492_ENG_HTML.php) and piRNA Bank (http://pirnabank.ibab.ac.in/request.html) and to identify homology piRNA. No more than one mismatch and E-value below 10^−4^ were set as a criterion. All small RNA data has been deposited into the NCBI Gene Expression Omnibus database (Database ID: GSE54610).

### Differential expression profile of miRNAs among three libraries

To compare the differentially expressed miRNAs in the three libraries of gonad, each identified miRNA read was normalized to the total number of miRNA reads in each library and multiplied by a million. If the normalized expression (NE) of a certain miRNA was lower than 1 in all three group, further differential expression analysis was conducted without this miRNA. Results of the Audic–Claverie test, Fisher exact test, and Chi-squared 2×2 test with a Bonferroni correction for multiple comparisons and a p-value <0.05 indicated a unique miRNA differentially expressed. After normalized miRNA read count, the log_2_fold-change and *p*-value were calculated from the NE data. We compared the statistical significance of miRNA expression between every two libraries. When the NE of a certain miRNA was zero in one group, we considered that it was sex-specific in another group. Among every two groups, when *p*-value <0.05, meanwhile log_2_fold-change >1.0 or <−1.0, a specific miRNA was designated as up-regulated or down-regulated. Scatter plots were used to demonstrate differentially expressed miRNA between every two groups. When the normalized expression of a certain miRNA was zero between two groups, we revised its expression value to 0.01. Expression heatmap of the yellow catfish miRNAs and hierarchical clustering for miRNAs were constructed by R version 3.0.2.

### Estrogen treatment

One-year-old male yellow catfish (mean weight 100±5 g) were selected for treatment. Hormone-treated group fish were intraperitoneal injected with 17α-ethinylestradiol (EE2, Sigma–Aldrich) at a dose of 10 µg/g body weight. EE2 were dissolved in dissolved in 5% ethanol/95% saline. Control group fish were injected with ethanol/saline solution alone. Each group of fish was reared under a natural photoperiod (approximately 14 h light/10 h darkness) in the laboratory facility where water was aerated and maintained at 26°C. At 12, 36, 72 and 96 hours post treatment, more than three fish testes of each group were sampled together and immediately stored in liquid nitrogen container for RNA extraction.

### Quantitative Real Time-PCR

Quantitative real-time PCR (qRT-PCR) with iTaq Universal SYBR Green Supermix (Bio-Rad) was performed to profile the expression level of arbitrarily selected miRNAs in three libraries, as previously described [26]. Briefly, 1 µg of total RNA was reverse transcribed by using NCode VILO miRNA cDNA Synthesis Kit (Invitrogen). All real-time reactions were run using the CFX96 Touch Real-Time PCR Detection System (Bio-Rad). Specificity of amplification for each reaction was analyzed by dissociation curves using CFX manager software (Bio-Rad). The expression of miRNAs was normalized to that of 5.8 s using the 2 (−delta delta C(T)) method [27]. Each experiment was performed in triplicate.

### Statistical analysis

For statistical analysis, data was shown as mean ± SD for three independent experiments. The data was assessed by the Student's t-test. A probability (P) of <0.05 was considered statistically significant.

## Results

### Small RNA analysis in three libraries

**Figure 1:**
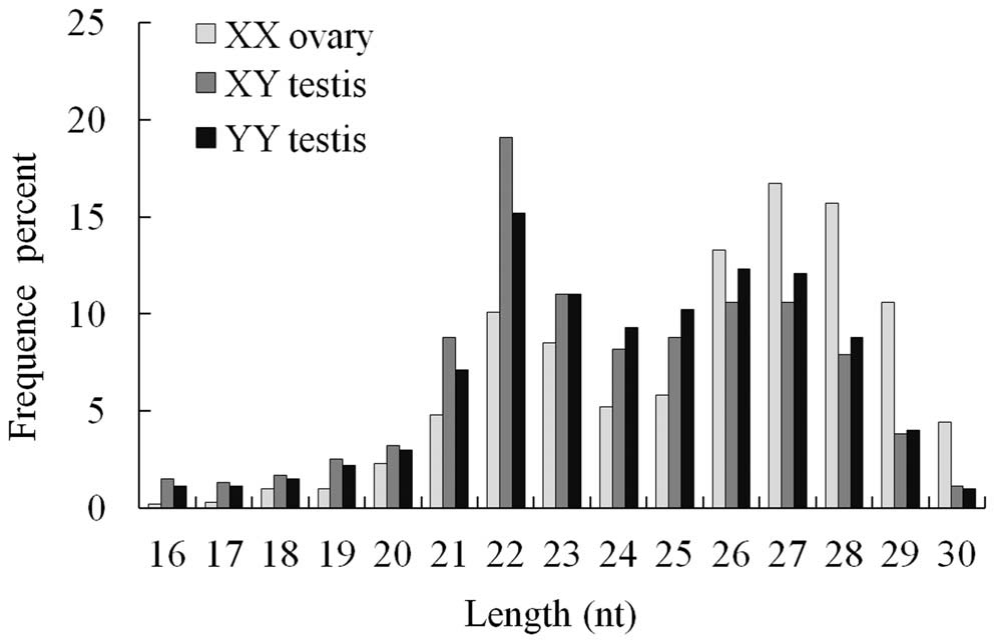
Length distribution of high quality reads in three sequenced small RNA libraries.

In order to identify sex-biased small RNA in yellow catfish, three small RNA (sRNA) libraries representing gonad of XX females, XY males and YY super-males were constructed with pooled total RNA and subjected to Solexa sequencing, respectively. A total of 35,873,807 raw reads were obtained from the three sRNA libraries. After removing adaptor sequences, junk reads and filtering length of sequences, 34,593,525 high quality reads were retained for mapping analysis (Table S1). Subsequently, we analyzed the length distribution based on distinct sequences to assess the sequencing quality (Figure 1). The length distribution of high quality reads was similar among the three libraries and displayed two peaks. One peak at 22–23 nt represents the typical size of Dicer-derived products. Another peak at 26–28 nt is corresponding to PIWI-interacting RNAs (piRNAs), which repress transposable elements and control animal gonads and germ line development [28]–[30].

After mapping analysis with ACGT101-miR v4.2 software, the high-quality sequencing sequences (sequ-seqs) could be assigned to several groups based on their hits (Table S2). In our data, certain known types of RNA sequences (e.g., mRNA, rRNA, tRNA, snRNA, snoRNA and repetitive sequence elements) were recognized by referring to Rfam and RepBase, and summarized respectively in Figure S1 and Table S3. The relative low proportion of unknown small RNA in data of mRNA, Rfam and RepBase indicated the high-quality of these libraries. Sequ-seqs that could not be mapped to zebrafish genome or their mapped sequence cannot form miRNA hairpin precursor were excluded from miRNA analysis, so that potential cell contamination will be eliminated for miRNA analysis. Due to lack of yellow catfish genome, more than half mapping reads were classified as nohit group, from which we found 500, 410 and 391 piRNA homology sequences, respectively corresponding to 44.3%, 35%, 34.7% of nohit group in XX, XY and YY sRNA libraries. Finally, 2,401,304 (XX ovary), 5,391,902 (XY testis) and 1,662,992 (YY testis) sequ-seqs were used for the following miRNA annotation and analysis.

### Characterization of miRNAs in yellow catfish gonad

To identify conserved miRNAs in the gonads of yellow catfish, we aligned the above collected sequ-seqs with currently known mature Deuterostoma miRNAs in miRBase allowing no more than one mismatches. There were 345 sequences mapped to the miRs and pre-miRs of selected species in the miRBase, and these pre-miRs were further mapped to the *Danio rerio* genome. In addition, another 94 sequences were mapped to the miRs and pre-miRs in miRBase, and the extended sequences could potentially form hairpins, whereas these 94 sequences mapped pre-miRs could not mapped to the *Danio rerio* genome. Totally, we identified 384 conserved miRNAs, of which 322, 372 and 348 miRNAs were expressed in XX ovary, XY testis and YY testis (Table S4).

Subsequently, we identified 113 novel miRNAs which are unmapped to any known pre-miRs but mapped to the *Danio rerio* genome and the corresponding extended genome sequences could potentially form hairpins structures (Table S4). Of the 113 novel miRNAs, 68, 82 and 82 unique miRNAs were distributed in XX ovary, XY testis and YY testis, respectively. In total, 497 unique miRNAs composed of 384 conserved miRNAs and 113 novel miRNAs were discovered in yellow catfish.

### Identification of sex-biased miRNA between ovary and testis

**Figure 2:**
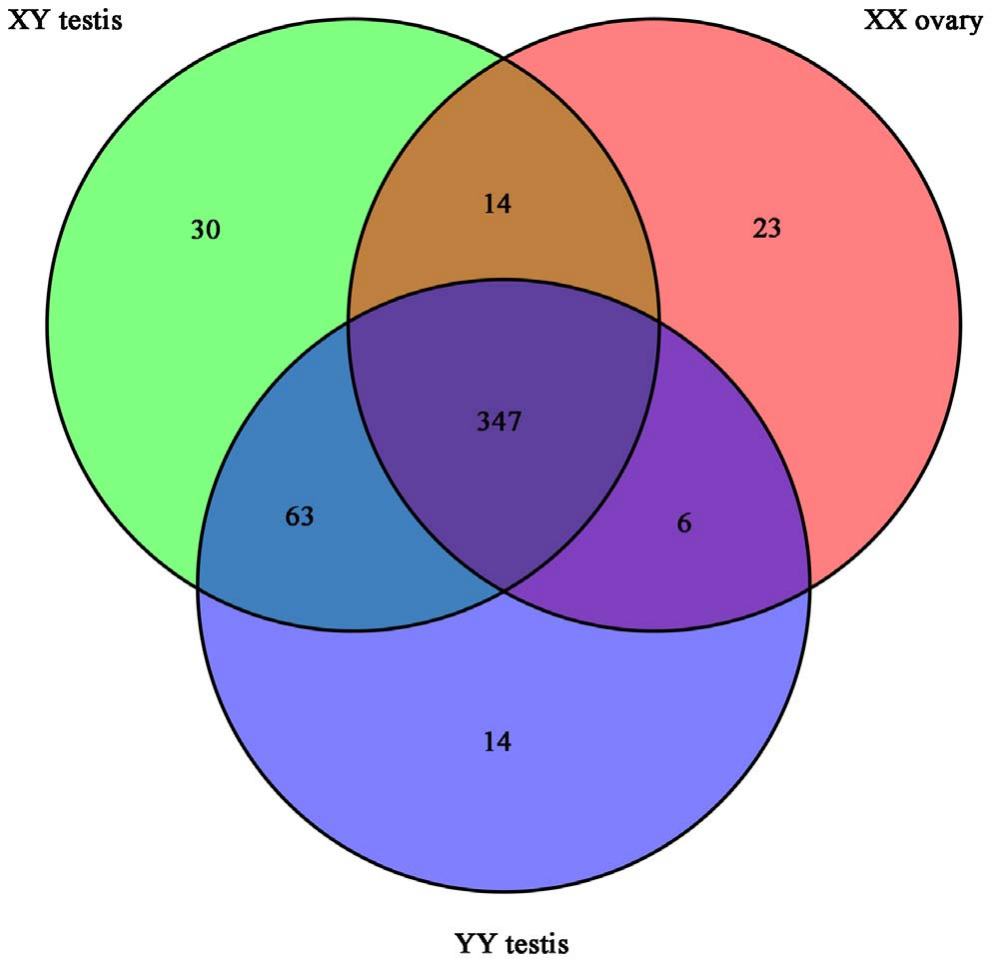
Venn diagram comparing the expression distribution of miRNAs in XX ovary, XY testis and YY testis. Numbers in parentheses represents number of co-expressed or differentially expressed miRNAs.

To identify sex-biased miRNAs that may play critical regulatory roles in sex differentiation of yellow catfish, we analyzed the distribution of miRNAs between XX ovary, XY testis and YY testis libraries (Figure 2). It was showed that 347 of 497 miRNAs (69.8%) were co-expressed in all three libraries, while 23 (4.6%), 30 (6%) and 14 (2.8%) miRNAs were only expressed in XX ovary, XY testis and YY testis, respectively (Figure 2 and Table S4). In addition, 63 miRNAs are specially expressed in both XY and YY testis, whereas there is no detection in XX ovary. The amount of miRNAs in XY testis (454, 91.3%) and YY testis (430, 86.5%) are higher than that in XX ovary (390, 78.5%), which implies that there are more conserved and novel miRNAs functioning in the male sex specification.

**Figure 3:**
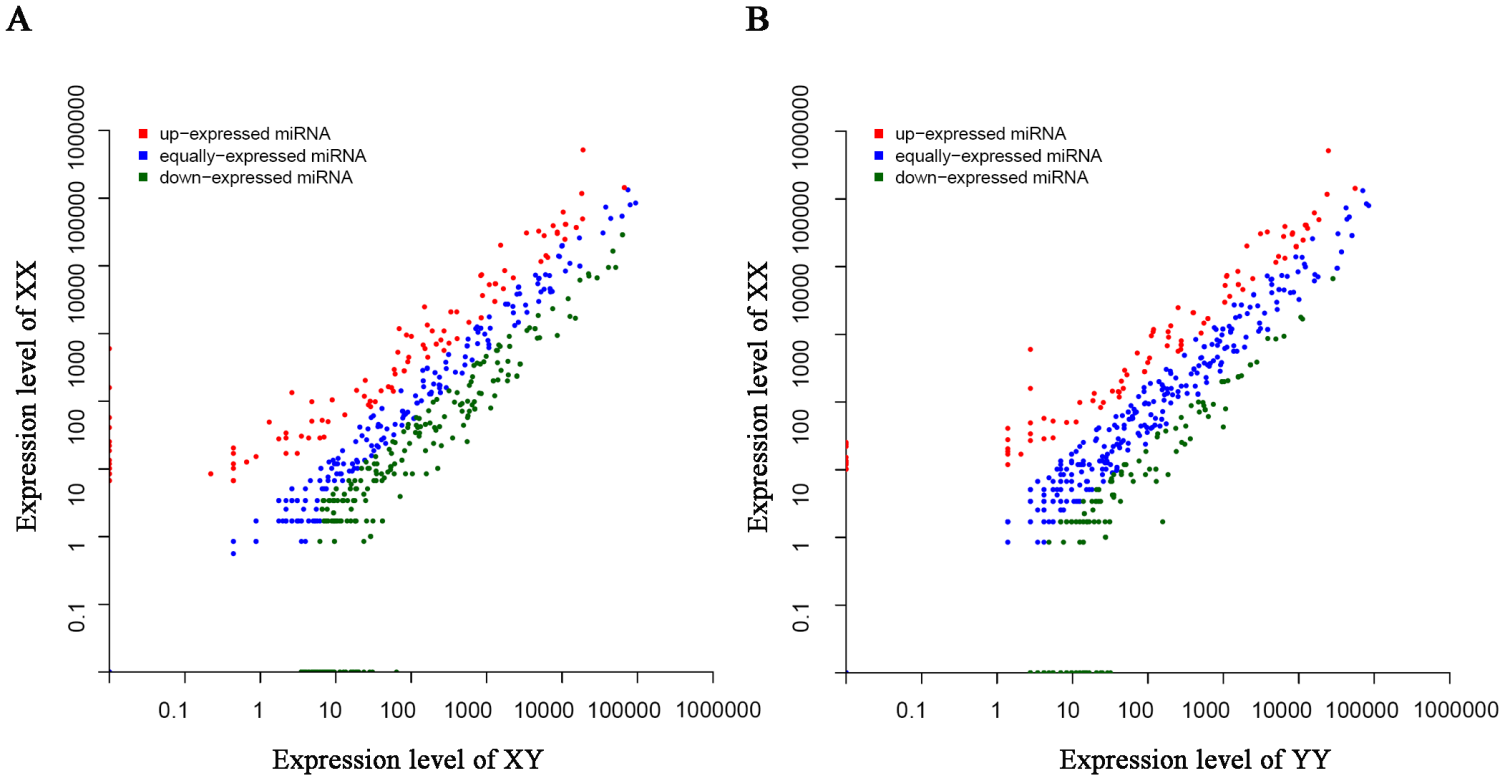
Difference of miRNA expression between ovary and testis. (A) Scatter plot of miRNA expression levels in XX ovary and XY testis. (B) Scatter plot of miRNA expression levels in XX ovary and YY testis. Each point represents a miRNA. The X and Y axes show the normalized expression (NE) of miRNAs in each gonad tissue.

We further compared the transcriptional level of co-expressing miRNAs between XY and XX library (XY/XX) or between YY and XX library (YY/XX), to define miRNAs related to testis or ovary differentiation (Table S5 and Figure 3). Analysis of 361 co-expressing miRNAs in XY/XX demonstrated that 204 miRNAs were significantly differentially expressed in both libraries. Of these 204 miRNAs, 144 and 60 miRNAs were up-regulated and down-regulated in the XY testis compared to the XX ovary. Also, we observed that 182 of 353 co-expressed miRNAs in YY/XX were significantly differentially expressed, with 128 miRNAs up-regulated and 54 miRNAs down-regulated in the YY testis compared to the XX ovary. In addition, we found three most abundant miRNAs (miR-146a, -21, -462) in XX ovary, and four most abundant miRNAs (miR-26a, -7g, -200a, -200b) in XY testis and YY testis. These sex-enriched miRNAs (except miR-26a in YY testis) had at least 2-fold difference in expression between male and female gonads. Moreover, seven dominant expression miRNAs in all three libraries are miR-100, -126a-3p, -202-5p, -30e, -143, -99, and 30d. It was noticeable that expression levels of miR-21-5p, miR-21-3p and miR-462-5p are more than 4-fold in XX ovary as compared to XY testis and YY testis (Table S5). Intriguingly, we found that miR-200 family had a male-bias expression in yellow catfish. The expression of six miR-200 members (miR-200a, -b, -c and their star sequences) in XY testis and YY testis are all significantly higher than XX ovary.

### miRNAs differentially expressed between XY testis and YY testis, and their potential roles for testis development

**Figure 4:**
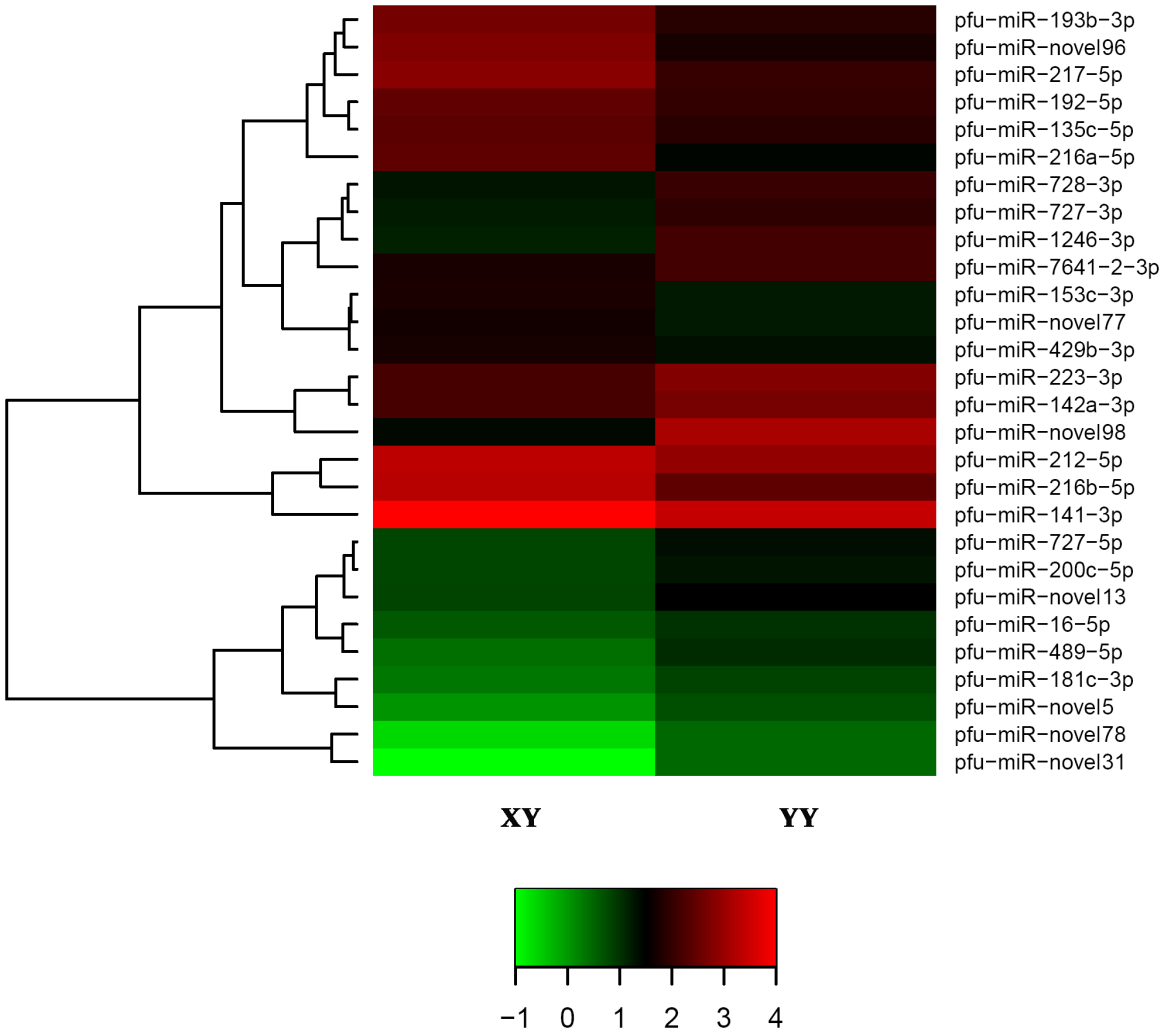
Hierarchical clustering of miRNAs differentially expressed between XY testis and YY testis. The heat map was drawn with log_10_NE of each miRNA. Green, black and red indicate low frequency, middle and high frequency miRNA cloned in the library. Color map is used to distinguish the difference of expression.

In Figure 2, we observed 43 and 20 miRNAs only expressed in XY or YY testis transcriptomes respectively, which suggests that testes with different genotypes own divergent miRNA expression patterns. Subsequently, we compared the expression levels of 410 co-expressed miRNAs between XY and YY testis (Table S5), and found a similar expression level for 93% co-expressed miRNAs, such as 5 dominantly expressed miRNAs, miR-26a, miR-7g, miR-200a, miR-200b and miR-103. Furthermore, 28 miRNAs were differently expressed, including 16 up-regulated and 12 down-regulated miRNAs in the YY library when compared with the XY library (Figure 4). Expression of several miR-200 family members, such as miR-141 and miR-429 were lower in YY testis than in XY testis.

**Figure 5:**
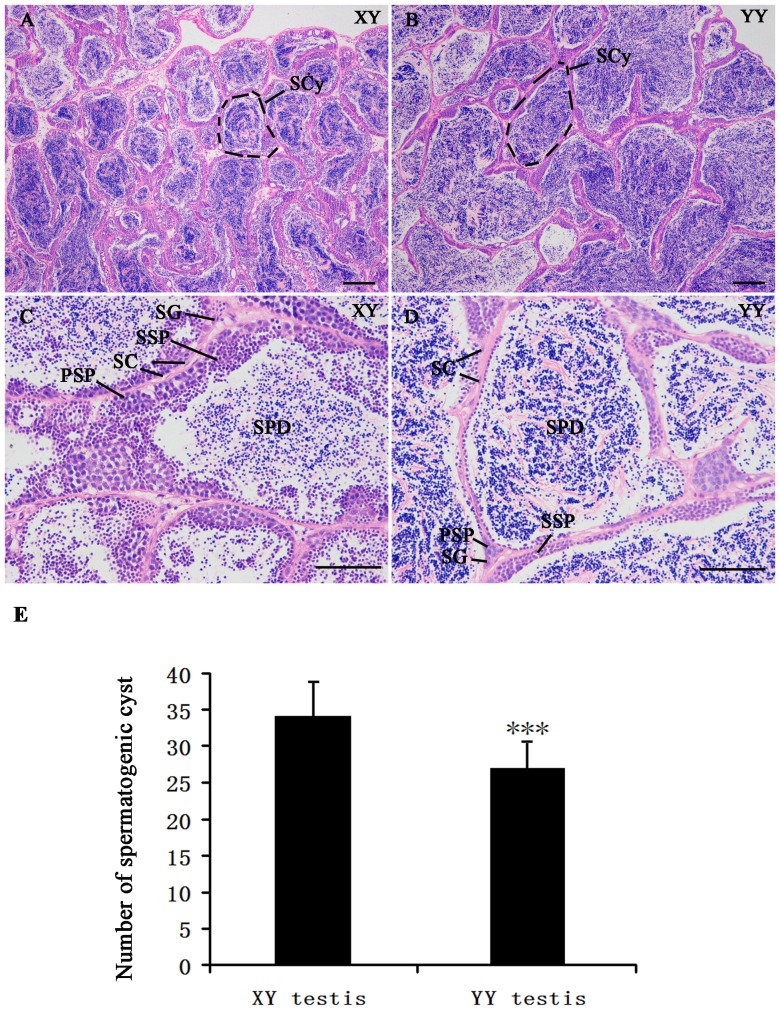
Histological section analysis of testis structure in yellow catfish with hematoxylin and eosin staining. (A–D) A and C—XY testis. B and D—YY testis. Scale bars: 200 µm (A and B), 100 µm (C and D). SC: sertoli cells, PSP: primary spermatocyte, SSP: second spermatocyte, SCy: spermatogenic cyst, SPD: spermatid, SG: spermatogonia. (E) Quantification of spematogenic cyst in XY testis and YY testis. The data represents number of spematogenic cyst in each figure at 200 µm scale bar (39 pictures for each tissue from three individuals). ***p<0.001.

Histological evaluation based on hematoxylin and eosin (HE) staining was performed on testis of XY and YY yellow catfish with similar size at one-year old (males usually mature about 2 years old) (Figure 5). In each field of vision under microscope, we figured out that spermatogenic cyst in YY testis is 25.8% larger than that in XY testis (P<0.001). Meanwhile, it was obvious that more spermatid and less spermatocyte in spermatogenic cyst of YY testis compared with XY testis, suggesting that the degree of testis maturity in YY super-male exceeds XY male yellow catfish. For the cystic mode of spermatogenesis in teleost fishes, germ cell number and volume greatly increase per cyst during the process of testis development [31], [32].

**Figure 6:**
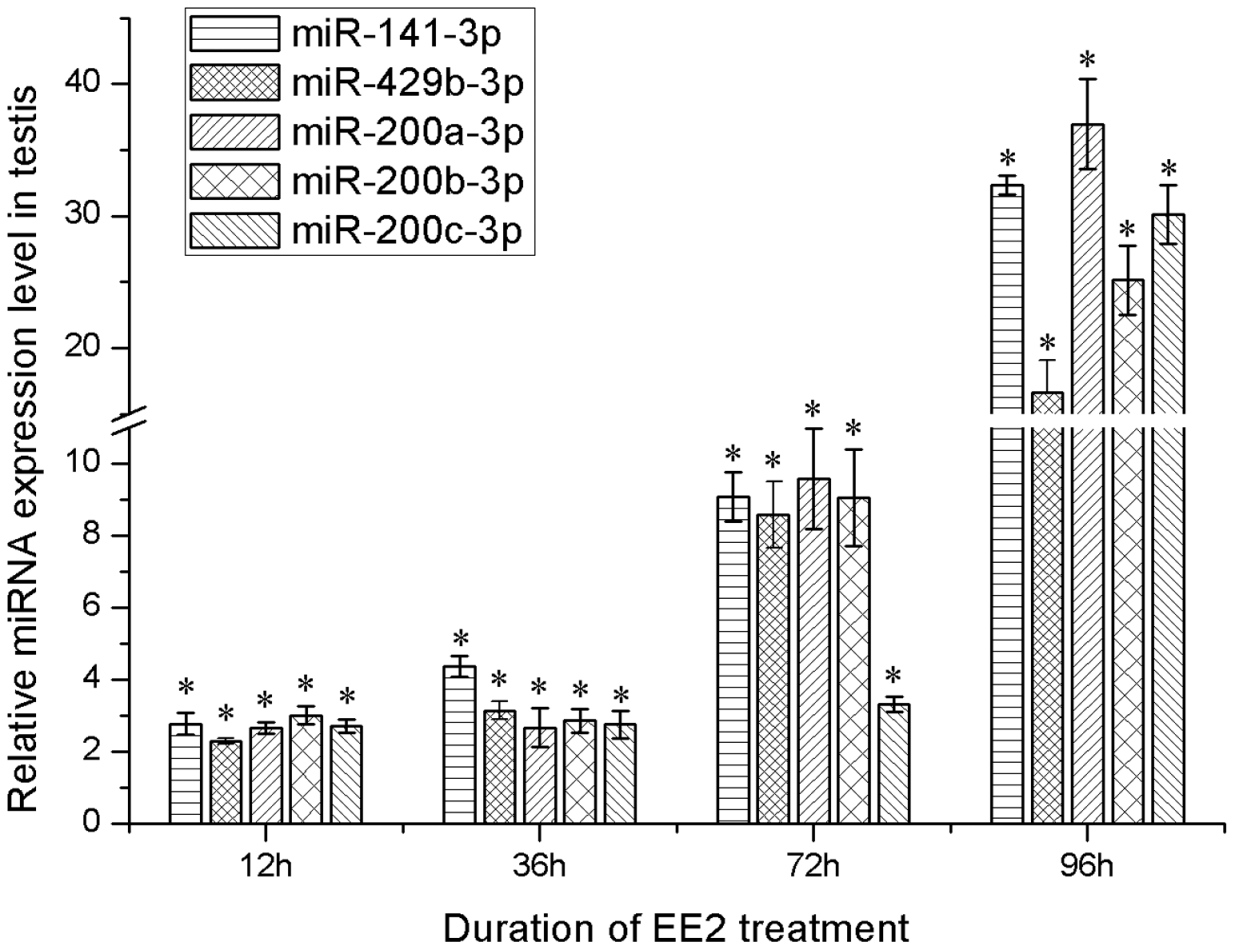
Relative expression of miR-200 family members in testis after EE2 treatment. The ratio of miRNAs to 5.8 s in control groups was set to 1 in every time point, and EE2 treated groups were normalized to this ratio of corresponding control group. *p<0.05.

In order to investigate whether miRNAs play some roles in testis development, we checked the expression of miR-200 family members in testis treated with high dose of estrogen that would impair testis development and cell proliferation [33]. Expression of five miR-200 family members (200a-3p, 200b-3p, 200c-3p, 141-3p, 429b-3p) was significantly up-regulated at 12 h, and were gradually rising to a higher value at 96 h after EE2 treatment compared to the expression of control group (Figure 6).

### Quantitative RT-PCR validation

**Figure 7:**
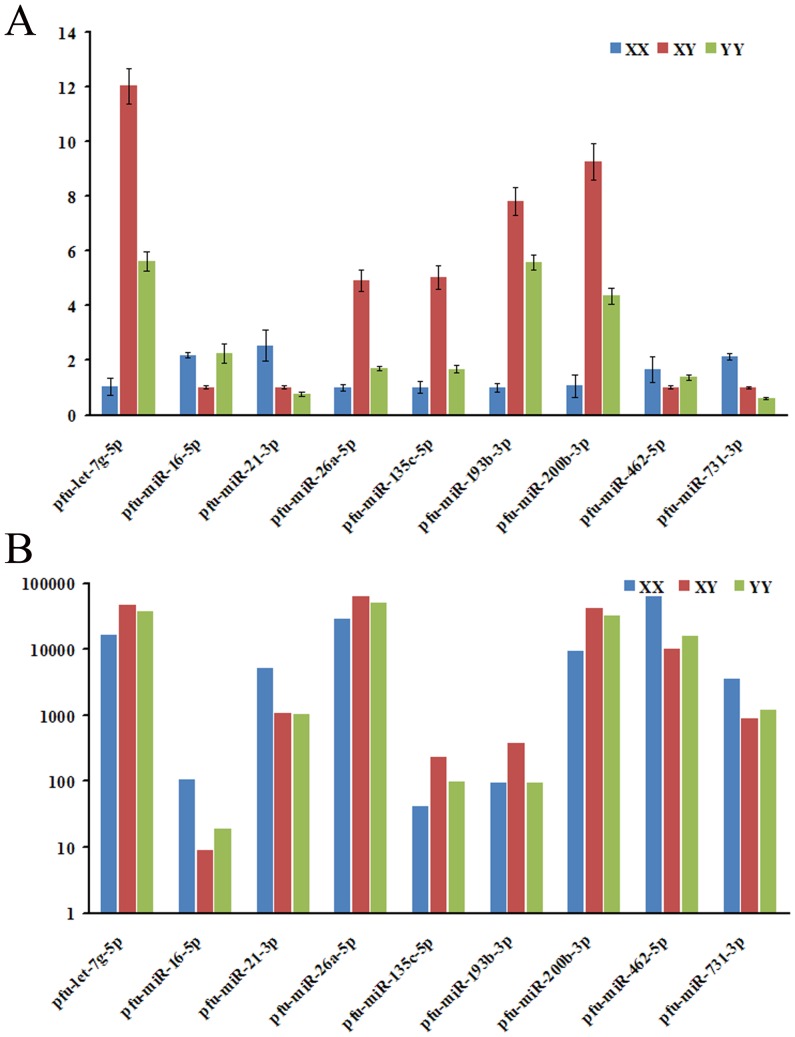
qRT-PCR validation of differentially expressed miRNAs between XX ovary, XY and YY testis with solexa sequencing technology. (a) Profile of relative expression of miRNAs by qRT-PCR; (b) Profile of sequencing frequencies for miRNAs with normalized expression (NE) data.

To verify the Solexa sequencing data, nine co-expressed miRNAs were randomly selected and determined using quantitative RT-PCR (qRT-PCR) (Figure 7). The nine miRNAs includes four miRs (miR-16-5p, miR-21-3p, miR-462-5p, miR-731-3p) relatively high expressed in XX and five miRs (let-7g-5p, miR-26a-5p, miR-135c-5p, miR-193b-3p, miR-200b-3p) relatively high expressed in XY. The relative expression levels of eight miRNAs were consistent with the Solexa sequencing results, except a slight inconsistency of miR-727-5p. It is possible that the primers used for qRT-PCR can bind miRNA species with a few mismatches that were not considered by the bio-informatics analysis.

## Discussion

Yellow catfish, one of the important economical fish in China, displays significant growth difference between male and female. Recently, YY super-male yellow catfish has been created and applied to massive all-male breeding by molecular assisted technology. To explore miRNAs that express differentially between different sexes and understand the roles of miRNA in gonad development, we characterized the gonad transcriptome of yellow catfish and identified 384 conserved miRNAs and 113 potential novel miRNAs.

Interestingly, there are more abundant reads of piRNAs homologue in XX ovary compared with XY and YY testis (Figure 1). Meanwhile, the proportion of piRNAs was higher in XX ovary than in XY and YY testis. Atlantic Halibut (*Hippoglossus hippoglossus*), a fish with an XY sex-determining system, also has a considerable enrichment of piRNAs in ovary compared with that in testis by SOLiD sequencing. However, there is no obvious typical miRNA peak in gonad of Atlantic Halibut [34], [35]. In the miRNA transcriptome of Nile Tilapia by Solexa sequencing, there are both typical miRNA and piRNA peaks [36]. Female enriched piRNAs have been revealed to be derived from W chromosome [37], suggesting that there may be more piRNA derived from X chromosome than Y chromosome in yellow catfish. tRNA-derived small RNAs have been revealed to be accounted for the majority of the 30–34 nt small RNA population that are enriched in mature mouse sperm, but not in adult testes and uterus [38]. However, the length distribution of analyzed reads in our libraries is 15–30 nt, and the dominant reads were 22–23 nt and 26–28 nt that represents miRNAs and piRNAs. These indicated that tRNA-derived small RNAs are not abundantly existed in our libraries constructed from immature gonads, and should be included in the types of tRNA sequences, not in nohit group (Figure S1).

In view of different expressed miRNA between XX ovary, XY and YY testis (Table S4), the expression pattern of some miRNAs between ovary and testis are consistent among several vertebrates. As in yellow catfish, miR-143 was also a dominant miRNA detected in both ovary and testis of bovine and porcine [11], [39]. We observed that miR-21-5p, miR-21-3p and miR-462-5p, the most abundant miRNAs in XX ovary that have more than 4-fold expression in XX ovary as compared to XY testis and YY testis (Table S5). Similar expression difference of miR-21 was also observed in ovary and testis of holstein cattle and adult mouse by solexa sequencing [11], [40]. MiR-21 has been widely known for its anti-apoptotic function in many cancers and been shown to block the apoptosis of granulose cells in ovary [41]. Besides, the expression of has-miR-21 was up-regulated when the mouse granulose cells and human endometrial stromal cells or glandular epithelial cells were treated with the ovarian steroids [42], [43]. MiR-462 has only been detected in fish species such as zebrafish, channel catfish and blunt snout bream [44]–[46]. Moreover, miR-462 was differentially expressed during vitellogenesis in zebarfish [45]. The expression level of some miRNAs in ovary are always correlated with their biological function during ovarian development [47]. Together, these findings suggested that miR-462 and miR-21 may play a particularly important role in ovary development.

YY super-male yellow catfish provides a unique model for fish testis development. HE histological evaluation shows that the degree of testis maturity in YY super-male exceeds XY male yellow catfish with same age. It was noticeable that miR-200 family members including miR-200a/b/c and their star sequences, miR-141 and miR-429/429a/429b all have a male-biased expression. Most miR-200 family members in yellow catfish have more normalized reads in XY testis than YY testis. Recent studies demonstrated that miR-141 is mainly expressed in the reproductive system, and gradually decreased during male germ cell development and in neonatal spermatogonia [48], [49]. In testicular tissue of asthenozoospermia and oligoasthenozoospermia patients, miR-141 and -429 were significantly increased compared with normozoospermic men [50]. Another study also detected up-regulation of miR-141 and -429 in seminal plasma of non-obstructive azoospermia patients compared with fertile controls. The expression profile of miR-141 and miR-429 was inversely associated with their methylation status [51]. All above data suggested that miR-141 and -429 are correlated with normal testis development and spermatogenesis in human.

Based on previous estrogen treatment studies, the dose of EE2 (10 ug/g body weight) was considered as a relative high concentration [33], [52], [53]. Under high dose of EE2 treatment, testis development and spermatogenesis is impaired, including inhibition of spermatogonia proliferation and differentiation, apoptosis of undifferentiated spermatogonia and reduction of seminal fluid volume [31], [54]–[56]. The increased expression of miR-200 family members in male testis were observed after estrogen treatment, indicating that miR-200 family might play a role in inhibiting spermatogenesis. Recent studies indicated that miRNAs play an important role in early male germ cell proliferation and late spermatogenesis [49], [57]. Hence, the relative low expression of miR-200 family members in YY testis may imply that their important function during testis development and spermatogenesis in yellow catfish. Identification of potential targeting gene and further functional studies are needed to examine the role of miR-200 family members and other differently expressed miRNAs between XY and YY testes. In addition, further characterization of these conserved miRNAs could contribute to a better understanding of the molecular mechanisms of miRNA in teleost gonad development.

## Supporting Information

Figure S1 Composition of non-coding RNAs mapped to RFam among the total sequence reads (A, B and C) and unique sequence reads (D, E and F) in the XX, XY and YY library, respectively.
(TIF)

Table S1 **Overview of reads from raw data to high quality reads.**
(XLS)

Table S2 **Group category of small RNA in the three libraries.**
(XLS)

Table S3 **Repeat sequence category of three small RNA libraries.**
(XLS)

Table S4 **Information of conserved miRNAs & novel miRNAs in three sequenced small RNA libraries.** NE = Actual miRNA count/Total count of clean reads * 1,000,000.
(XLS)

Table S5 **Abundance and differential expression of co-expressed miRNAs in every two libraries.** Fold change = log2 (sample 1 NE/sample 2 NE); Expression Level: High (The number of reads in the following reported miRNAs is higher than the average copy of the data set), Middle (The number of reads in the following reported miRNAs is higher than 10 and less than average copy of the data set), Low (The number of reads in the following reported miRNAs is less than 10).
(XLS)
