# Supplementary figures and images for: Sex-Biased miRNAs in Gonad and Their Potential Roles for Testis Development in Yellow Catfish
Source: PLoS One. 2014 Sep 17;9(9):e107946. doi: 10.1371/journal.pone.0107946 (PMC4168133; doi:10.1371/journal.pone.0107946)

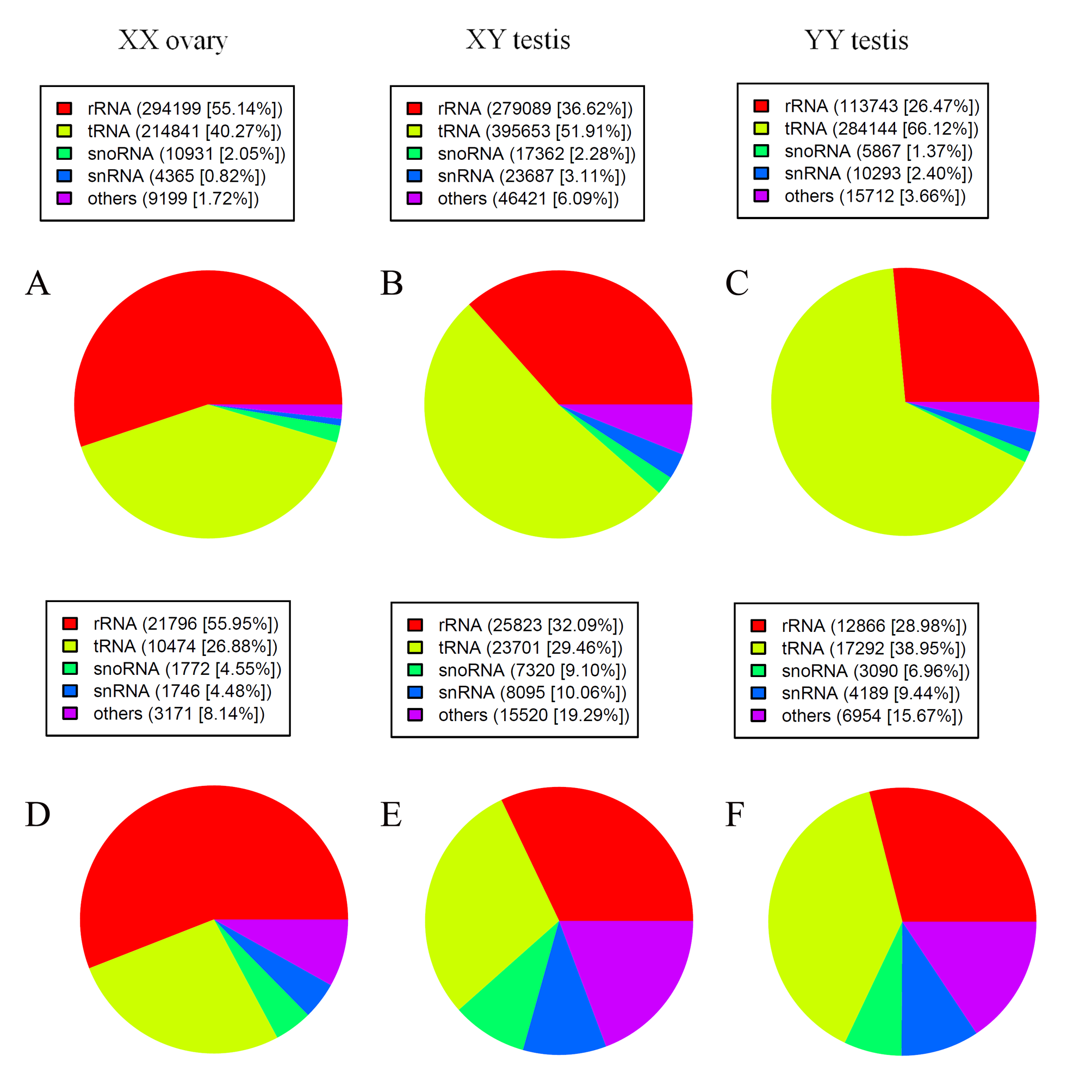

Supplement: Figure S1 — Composition of non-coding RNAs mapped to RFam among the total sequence reads (A, B and C) and unique sequence reads (D, E and F) in the XX, XY and YY library, respectively. (TIF) [file pone.0107946.s001.tif]
